# Urine-based point-of-care testing for factor-Xa-inhibitors in acute ischemic stroke patients: a feasibility study

**DOI:** 10.3389/fneur.2023.1330421

**Published:** 2023-12-15

**Authors:** Thorsten R. Doeppner, Linus Olbricht, Toska Maxhuni, Omar Alhaj Omar, Ulrich J. Sachs, Martin B. Juenemann, Hagen B. Huttner, Stefan T. Gerner

**Affiliations:** ^1^Department of Neurology, University Hospital Giessen, Giessen, Germany; ^2^Center for Mind, Brain and Behavior (CMBB), Philipps University Marburg and Justus Liebig University Giessen, Giessen, Germany; ^3^Translational Neuroscience Network Giessen, Justus Liebig University Giessen, Giessen, Germany; ^4^Department of Neurology, University of Göttingen, Göttingen, Germany; ^5^Division of Haemostaseology, University Hospital Giessen, Giessen, Germany

**Keywords:** point-of-care test, direct oral anticoagulant, factor-Xa-inhibitor, anticoagulation, ischemic stroke

## Abstract

**Introduction:**

Direct oral anticoagulants (DOACs) have become widely used in clinical practice for preventing thromboembolic events. Point-of-care testing methods, particularly those based on urine samples, offer a promising approach for rapid and accurate assessment of DOAC presence. This pilot study aims to evaluate the utility of a urine-based DOAC dipstick test as a point-of-care tool for identifying DOAB presence in acute ischemic stroke (AIS) or transient ischemic attack (TIA) patients.

**Patients and methods:**

This prospective pilot study included patients with AIS/TIA eligible for DOAC-measurement. After exclusion of 3 patients, 23 patients with DOAC-intake (DOAC group; factor-Xa-inhibitors; *n* = 23) and 21 patients without DOAC-intake (control-group) remained for analyses. The urine-based DOAC dipstick test and parallel blood-based specific DOAC-level assessment were performed in all patients. Time-intervals of sampling urine/blood sampling and result of DOAC-test were recorded to analyze a potential time benefit based on dipstick evaluation.

**Results:**

The urine-based DOAC dipstick test demonstrated high sensitivity (100%) and specificity (100%), correctly identifying all patients with anticoagulatory activity due to DOAC intake (i.e., anti-Xalevel ≥30 ng/mL). Moreover, the visual readout of the test provided semiquantitative information on drug-specific anti-Xa levels, showing a sensitivity of 83% and specificity of 93% to detect anti-Xa levels ≥120 ng/mL. The dipstick test exhibited a median time-benefit of 2:25 h compared to standard blood-based DOAC-level testing.

**Discussion:**

The results of this pilot study underline the efficacy of urine-based point-of-care testing as a rapid and reliable method for assessing DOAC presence in patients with acute ischemic stroke.

**Conclusion:**

The value of this tool for clinical decision-making in stroke management needs to be established in future trials.

**Clinical Trial Registration**: Clinicaltrails.org identifier [NCT06037200].

## Introduction

Direct oral anticoagulants (DOAC) have emerged as the preferred choice for oral anticoagulation (OAC), primarily attributed to their by half reduced risk for intracranial bleeding compared to vitamin-K antagonists (VKA) (1–4). The widespread utilization of DOACs presents a challenge in emergency care, as standard coagulation assessment methods do not accurately reflect the anticoagulatory effect of these agents (5–7).

In patients with acute ischemic stroke (AIS), the use of DOACs remains a contraindication for intravenous thrombolysis according to international guidelines, owing to the increased risk of bleeding complications in these patients (8, 9). However, drug-specific DOAC-levels were reported to guide decision-making for recanalizing therapies in these patients (3, 8, 10–13). Yet, the availability of specialized tests for DOAC monitoring is currently limited, and their prolonged turnaround times may lead to critical delays in time-sensitive treatments, such as hemostatic reversal in patients with intracerebral hemorrhage or intravenous thrombolysis in acute ischemic stroke (9, 14).

Several studies have shown the time-saving benefit of point-of-care (POC) testing in the setting of acute stroke (15–17). For patients with VKA, POC is already available and offers safe identification of patients with relevant anticoagulatory activity in order to guide clinical decision-making (16). In the case of DOAC patients, a urine-based dipstick test has shown promising results in detecting and excluding anticoagulatory activity of DOACs in an unspecific population of emergency care unit patients (18). Hence, data on stroke patients are scarce.

In this context, we present the results of a pilot study investigating the utility of a urine-based DOAC dipstick test as a POC tool in patients with AIS or transient ischemic attack admitted to a certified stroke unit of a university hospital in Germany. The primary objectives of this pilot study were to evaluate the sensitivity and specificity of the urine-based dipstick test in detecting DOAC presence and to explore its potential for quantitative analysis of drug-specific DOAC levels. Additionally, we sought to investigate the potential time-saving benefit of POC testing using the DOAC dipstick compared to traditional blood-based specific DOAC level assessment.

## Materials and methods

### Patient selection

In this prospective study, we recruited patients treated for acute ischemic stroke or transient ischemic attack at the certified stroke-unit of the Department of Neurology, University Hospital Giessen, Germany, over a period of 7 months from January to July 2023. Patients were eligible for inclusion if either they had a safe intake of DOACs certified by their electronic patient chart and treating nurse within the last 24 h, or if they had not reported taking any DOAC within the last 7 days (control group). All patients who participated in the study provided informed consent. Patients below the age of 18 or those who lacked the ability to provide informed consent were excluded from the study. The study was approved by the local ethics committee and institutional review board at Justus-Liebig University, Giessen, Germany, with the reference number AZ 194/22.

### Clinical parameters

We collected data on various clinical parameters, including demographic information (age, sex), prior medical history (such as arterial hypertension, renal impairment, and use of platelet function inhibitors or statins), clinical presentation on admission (measured using the National Institutes of Health Stroke Scale), length of stay at the stroke-unit, and stroke characteristics [including the rate of intravenous thrombolysis (IVT), endovascular therapy (EVT), and stroke location]. The clinical status of patients at discharge was assessed using the modified Rankin scale (mRS) (19). For patients taking DOACs, we also recorded the specific DOAC agent, dosage, and time since the last intake. We documented cases where patients received a reduced dose of DOAC, defined as less than 300 mg dabigatran, 20 mg rivaroxaban, 10 mg apixaban, or 60 mg edoxaban per day (20). Additionally, we assessed the indication for oral anticoagulant therapy (OAC).

### Coagulation assessment

Coagulation assessment was performed in all patients using both blood and urine tests. For blood coagulation assessment, venous blood samples were collected from each patient and processed following standard laboratory protocols. Hemostatic parameters were measured, including international normalized ratio (INR), platelet count, and activated partial thromboplastin time (aPTT) using automated coagulation analyzers. All DOAC and control patients underwent additional testing for drug-specific anti-Xa levels, as there was no patient with intake of the thrombin-inhibitor Dabigatran.

Additionally, coagulation assessment was carried out using the Doasense™ dipstick, a novel POC device designed for the detection of Direct Oral Anticoagulants (DOACs) in urine and approved in the European union, as reported elsewhere (18, 21). The dipstick is designed for detection of DOACs at a level ≥ 30 ng/mL, providing a visual semi-quantitative result of either negative, single positive (+ or ++), or double positive (++) based on the concentration of DOACs in the sample. Furthermore, semi-automatic readout of the Dipstick was conducted by the Doasense Reader, for further details please see (22). The dipstick results were acquired by physicians who had undergone online training (provided by Doasense™, available under www.doasense-training.com) in coagulation assessment utilizing the DOAC dipstick and were blinded to the patient’s history of DOAC-intake. Results of coagulation testing, time-points of blood and urine sampling as well as time-point of the test-result were recorded.

### Outcome measures

Primary endpoint was the sensitivity of the Doasense dipstick to detect relevant anticoagulatory activity, defined as drug-specific anti-Xa level of ≥30 ng/mL, in patients with factor-Xa inhibitors (7, 23).

Secondary endpoints comprised the specificity of the Doasense dipstick to rule out relevant anticoagulatory activity, as well as time-intervals between sampling of blood or urine and testing result, i.e., anti-Xa activity or Doasense Dipstick (visual and automatic result), respectively.

### Statistical analyses and predefined subgroups

The study outcomes were analyzed using statistical software (SPSS, IBM SPSS Statistics 28.0), and graphical illustrations were created using Adobe Illustrator (Adobe, Adobe Illustrator 2023). Baseline characteristics were analyzed by dividing patients into two groups: those with recorded DOAC intake and those without DOAC intake. Descriptive statistics, including mean (standard deviation) for normally distributed data, median (interquartile range) for non-normally distributed data, and absolute numbers (percentage) for nominal data, were provided.

We performed graphical analyses of predefined subgroups, i.e., DOAC-patients categorized into (i) normal versus reduced dose of DOAC and (ii) time since the last DOAC-intake (<3 h, 3–12 h, and > 12 h). Furthermore, we conducted receiver operating characteristics (ROC) analysis to investigate the association between specific anti-Xa levels and the visual result of the DOAC dipstick test (24). The optimal drug-specific anti-Xa cut-off value was identified to distinguish between (i) negative results and at least single positive results (i.e., negative versus + or ++) on the DOAC dipstick and (ii) between negative or single positive results and double positive results (i.e., negative or + versus ++) on the DOAC dipstick. Sensitivity, specificity, and Youden index with 95% confidence intervals were calculated for each identified anti-Xa threshold level (23, 25).

## Results

Overall, 47 patients with treated acute ischemic stroke or transient ischemic attack at a certified stroke-unit were recruited over 10 weeks of active enrollment between 01 and 07/2023. After excluding three patients (two with missing urine samples and one with a missing blood sample), the final analysis was conducted on 44 patients. Among them, 23 AIS-patients had intake of DOAC (all factor-Xa inhibitors), whereas 21 patients did not have DOAC intake in the last 7 days and served as the control group.

### Patients’ and DOAC characteristics

The clinical characteristics of DOAC- and control patients are provided in Table 1. Mean age was 78.5 years in the DOAC group and 67.6 years in the control group, respectively. Prior renal impairment was present in 4 (17.4%) patients in the DOAC group but none in the control group. More than half of the patients in both groups experienced transient symptoms, with a median NIHSS score of 1 (interquartile range: 0–3) at presentation.

**Table 1:** Characteristics of included patients.

Patients with AIS (*n* = 44)	DOAC (*n* = 23)	Control (*n* = 21)
Age, y; mean (SD)	78.5 (9.5)	67.6 (13.2)
Female sex; n (%)	9 (39.1)	8 (38.1)
Prior medical history		
Premorbid mRS; median (IQR)	1 (0–1)	0 (0–0)
Arterial hypertension; n (%)	21 (91.3)	14 (66.7)
Peripheral arterial occlusive disease; n (%)	2 (8.7)	0 (0)
Renal impairment; n (%)	4 (17.4)	0 (0)
Comedication; n(%)		
Platelet function inhibitor	1 (4.3)	19 (90.5)
Statin	22 (95.7)	20 (95.2)
Stroke characteristics		
Acute ischemic stroke; n (%)	10 (43.5)	10 (47.6)
Transient ischemic attack; n (%)	13 (56.5)	11 (52.4)
NIHSS on admission; median (IQR)	1 (0–4)	1 (0–3)
Intravenous thrombolysis; n (%)	1 (4.3)	6 (28.6)
Endovascular thrombectomy; n (%)	4 (17.4)	1 (4.8)
Left hemispheric symptoms; n (%)	9 (40.9)	7 (33.3)
Length of stay at stroke-unit, d; median (IQR)	5 (4–12)	4 (5–8)
mRS at discharge; median (IQR)	1 (0–3)	0 (0–1)

Provided are the baseline characteristics of patients dichotomized into DOAC intake and control group. Data are presented as mean (± standard deviation), median (interquartile’s range) or absolute number (percentage). DOAC, direct oral anticoagulant; SD, standard deviation; y, years; mRS, modified Rankin scale (ranging from 0, no symptoms, to 6, dead); NIHSS, National Institute of Health Stroke Scale (ranging from 0 to 42 with higher scores indicating more severe symptoms); d, days.

Among the patients with DOAC intake (*n* = 23; see Table 2), Apixaban was the most frequently used DOAC agent (*n* = 17, 73.9%), followed by Rivaroxaban (*n* = 4; 17.4%) and Edoxaban (*n* = 2; 8.7%). The primary indication for DOAC use was the prevention of thromboembolism due to atrial fibrillation (91.3%). Reduced DOAC dosage was used in 6 of the 23 patients (26.1%).

**Table 2:** Characteristics of DOAC intake.

Patients with DOAC-intake (*n* = 23)	DOAC (*n* = 23)
DOAC-agent, n (%)	
Rivaroxaban	4 (17.4)
Apixaban	17 (73.9)
Edoxaban	2 (8.7)
Reduced dose	6 (26.1)
Last intake of DOAC, h; median (IQR)	4 (3–7)
Indication for OAC; n(%)	
Atrial fibrillation	21 (91.3)
Thrombosis	0 (0)
Thromboembolism	0 (0)
Other	2 (8.7)

For patients with DOAC-intake only (*n* = 23), information about DOAC-agent, last intake, dosing and indication for oral anticoagulation are shown. Reduced dose was defined as daily dose of less than 20 mg rivaroxaban, 10 mg apixaban, or 60 mg edoxaban. DOAC, direct oral anticoagulant; OAC, oral anticoagulation; h, hours.

### Coagulation testing and dipstick results

Standard coagulation parameters were not significantly altered in both groups, as presented in Table 3. The median drug-specific anti-Xa level in the DOAC group was 120 (interquartile range: 60–170) ng/mL, with 21 patients (91.3%) showing relevant anticoagulatory activity.

**Table 3:** Coagulation assessment and procedural times.

Patients with AIS (*n* = 44)	DOAC (*n* = 23)	Control (*n* = 21)
Laboratory measurements		
INR; median (IQR)	1.1 (1.0–1.2)	1.0 (1.0–1.1)
aPTT, s; median (IQR)	33 (29–37)	27 (26–32)
Thrombocyte count, 10*9/L; median (IQR)	205 (166–266)	240 (215–289)
Estimated GFR, mL/min; median (IQR)	75 (63–102)	100 (78–115)
Specific anti-Xa levels		
Specific Anti-Xa activity, ng/mL; median (IQR)	120 (60–170)	0 (0–0)
Specific Anti-Xa activity ≥30 ng/mL; n (%)	21 (91.3)	0 (0)
Dipstick results; n(%)		
Automatic result positive	21 (91.3)	0 (0)
Visual result		
Creatinine	23 (100)	21 (100)
Factor-Xa inhibitor negative	2 (8.7)	21 (100)
Factor-Xa inhibitor +	9 (39.1)	0 (0)
Factor-Xa inhibitor ++	12 (52.2)	0 (0)
Sensitivity, %	100	n/a
Specifity, %	100	100
Time intervals		
Blood sample to result, min; median (IQR)	203 (108–302)	127 (73–282)
Urine sample to result, min; median (IQR)	17 (15–18)	15 (13–16)

Coagulation parameters and time-delay of coagulation assessment are provided in patients dichotomized into DOAC intake and control group. Data are presented as median (interquartile’s range) or absolute number (percentage). DOAC, direct oral anticoagulant; IQR, interquartile’s range; INR, international normalized ratio; aPTT, activated partial thromboplastin time; GFR, glomerular filtration rate.

Urine collected by catheterization was used in 15/44 patients (34.1%) and spontaneous urine was provided by 29/44 (65.9%) patients. The DOAC dipstick test correctly identified all patients with relevant anticoagulatory activity (21 out of 44 patients in total), both through automatic and visual readouts (9/12 with a single/double positive result), resulting in a sensitivity of 100%. The DOAC dipstick test also showed a specificity of 100%, as all patients with anti-Xa levels below 30 ng/mL tested negative. The result of urine-based DOAC testing was available median 15 min (interquartile’s range: 14–17 min) after begin of urine sampling in the overall cohort. Compared to standard blood-based anti-Xa level testing, which results were available after 160 min (interquartile’s range: 87–290 min), we observed a potential median time benefit of 145 min by urine based urine-based testing.

### Subanalyses of visual dipstick results and DOAC characteristics

**Figure 1:**
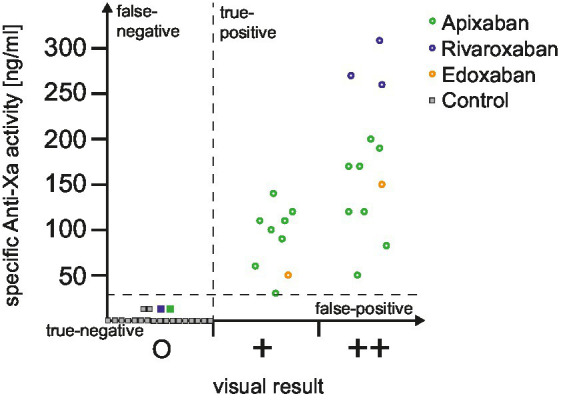
Distribution of drug-specific anti-Xa levels according to dipstick result. The distribution of drug-specific anti-Xa levels is depicted for all included patients, categorized based on the visual dipstick result. Circles represent a positive result obtained from the DOAC dipstick, while squares indicate a negative result. To improve clarity, only a subset of control patients is depicted in this figure for illustrative purposes (control patients not illustrated in this figure had all anti-Xa levels of 0 mg/mL). Additionally, dotted lines demarcate the area corresponding to true positive, false positive, true negative, and false negative results. DOAC, direct oral anticoagulant.

**Figure 2:**
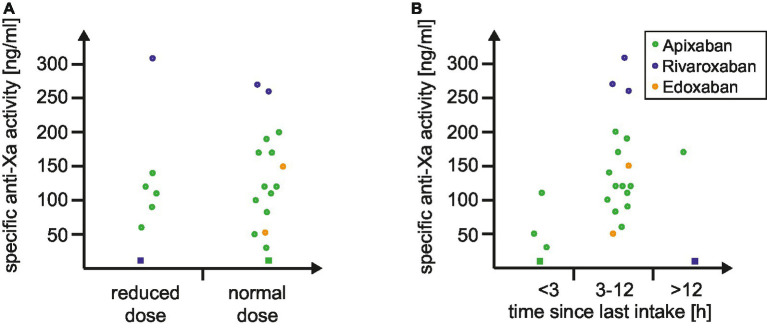
Distribution of specific anti-Xa levels according to **(A)** DOAC dosing and **(B)** time since last intake of DOAC. The distribution of drug-specific anti-Xa levels is depicted based on the dosing of DOAC **(A)** and the time since the last DOAC intake **(B)**. Circles represent a positive result by the DOAC dipstick, while squares indicate a negative result. Abbreviation: DOAC indicates direct oral anticoagulant.

In addition to the automated and dichotomized read-out of dipstick we explored if the visual analysis harbors add-on value in clinical routine. The distribution of drug-specific anti-Xa levels according to the visual dipstick result is illustrated in Figure 1. No false-negative or false-positive results were observed during our study. Median drug-specific anti-Xa levels were 100 (IQR 55–115) ng/mL in single positive and 170 (IQR 120–245) ng/mL in double positive tested patients. Subanalyses according to reduced versus normal DOAC-dosage (Figure 2A) and to time of last intake of DOAC (Figure 2B) revealed no concerns regarding the sensitivity of the urine-based testing in these subgroups.

### Quantitative assessment of anti-Xa levels via visual dipstick readout

**Figure 3:**
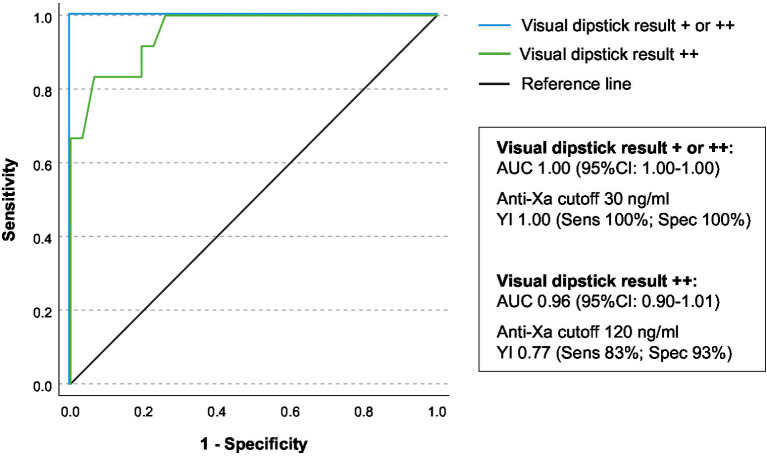
Association of drug-specific anti-Xa-levels and visual dipstick results. The receiver operating characteristic (ROC) analysis was conducted to assess the relationship between drug-specific anti-Xa levels and the visual result of the DOAC dipstick (+/++ illustrated as blue line, ++ illustrated as green line). The area under the curve (AUC) with the corresponding 95% confidence interval is presented. The Youden-Index, sensitivity, and specificity were calculated for both identified anti-Xa thresholds.

The ROC-analysis of anti-Xa levels (Figure 3) with the visual dipstick result demonstrated a true positive association, with an area under the curve (AUC) of 1.00 for at least single positive visual results and an AUC of 0.96 for double positive results by DOAC dipstick. The optimal threshold for detecting at least a single positive visual result was identified at anti-Xa levels of 30 ng/mL, and for detecting double positive results on the dipstick, the threshold was 120 ng/mL. The double positive dipstick results had a sensitivity of 83% and a specificity of 93% in detecting anti-Xa levels of at least 120 ng/mL (Figure 3).

## Discussion

In the present mono-center prospective pilot study, POC testing via urine dipstick enabled reliable and rapid identification and exclusion of relevant anticoagulatory activity in acute ischemic stroke patients with factor-Xa-inhibitor intake. In line, visual readout of the dipstick results permitted quantitative assessment of anti-Xa levels. Some aspects deserve special attention.

First, this study revealed a remarkable accuracy of the urine-based dipstick, achieving a 100% discrimination rate between relevant (≥30 mg/nL) anticoagulant activity and no anticoagulant activity, among patients taking factor Xa inhibitors. This finding is consistent with previous research exploring Point-of-Care (POC) testing using the DOAC dipstick. Previous investigations into the DOAC dipstick have reported sensitivities of 97% for detecting relevant anticoagulatory activity (≥30 ng/mL) in both outpatient clinic settings and emergency department patients (18, 26). Additionally, a separate study focusing on acute stroke patients presenting in the emergency room (*n* = 17) demonstrated that the DOAC-dipstick correctly identified 95% of patients with DOAC plasma levels >30 ng/mL (27), however, data on clinical and DOAC characteristics were not available for this cohort. Given the potential time-saving advantages of this approach in the context of acute stroke, it appears justifiable to extend the investigations to acute stroke patients presenting in the early-time window to determine whether this time-saving benefit may lead to improved outcomes (28, 29). Moreover, it is essential to recognize that many hospitals lack access to round-the-clock testing of specific DOAC levels, particularly with rapid turnaround times. In such settings, the DOAC dipstick could present a convenient and time-effective alternative in patients with suspected DOAC intake and may help for clinical-decision making regarding recanalizing therapies (11).

Second, the visual results obtained from the dipstick have demonstrated a promising potential for semi-quantitatively analyzing DOAC concentrations in urine, thus correlating with blood-based drug-specific anti-Xa levels. This aspect is of significant importance as it has the potential to improve decision-making for acute treatment of stroke patients. In contrast to the EHRA-recommended threshold of 30 ng/mL for anti-Xa levels in overall DOAC patients (7), studies focusing exclusively on DOAC patients with ischemic stroke or intracerebral hemorrhage have reported different thresholds (13). Notably, one study indicated that intravenous thrombolysis may also be feasible in patients with rivaroxaban intake that display a specific anti-Xa levels up to 100 ng/mL, considering the individual benefit–risk ratio (12). For patients with intracerebral hemorrhage (ICH), a cut-off value of 118 ng/mL was reported to be associated with an increased risk of hematoma enlargement (30). Consequently, the visual readout from the dipstick may offer valuable supplementary information to assist in clinical decision-making during these challenging scenarios including the detection of patients at high risk for recurrence of stroke (31).

Third, in the context of AIS, time plays a critical role (28). Prompt and accurate identification of DOAC usage is crucial to avoid delays in administering appropriate recanalizing therapies, particularly intravenous thrombolysis (28, 29, 32). Our study revealed that the urine-based dipstick test provided results within a median time of 17 min, leading to a significant time-benefit of over 2 h when compared to standard blood-based anti-Xa level testing. This rapid turnaround time has the potential to expedite decision-making in time-sensitive situations, thereby contributing to improved patient outcomes. Subanalyses based on visual dipstick results and DOAC characteristics further validated the reliability of this urine-based testing method in stroke patients. Furthermore, the dipstick test remained effective in detecting DOAC presence, irrespective of reduced versus normal dosage or the time since the last DOAC intake. These findings underscore the robustness of the dipstick test across various patient profiles and treatment scenarios (4, 33).

Despite these promising results, it is essential to acknowledge several limitations in this study. Notably, the sample size was relatively small, necessitating larger-scale studies to validate and extrapolate our findings to a more diverse patient population. Besides, while the urine-based dipstick test shows promise as a reliable DOAC detection tool, its performance in specific subgroups, such as patients with renal impairment, requires further investigation to ensure its applicability across various clinical scenarios. It is also important to note that our study was conducted in a stroke unit setting, and as such, the translation of our findings to the acute setting in an emergency room may be subject to certain limitations and would require additional evaluation. Moreover, this study did not routinely assess the duration until urine sampling or the percentage of patients unable to promptly provide urine samples. Consequently, the feasibility of urine-based POCT in the acute setting remains unknown.

## Conclusion

In conclusion, the results of this study underscore the efficacy of urine-based point-of-care testing in rapidly and accurately assessing the presence or absence of factor-Xa-inhibitor activity. Its potential for quantitative analysis needs to be validated by further research and larger-scale studies. Additionally, it is imperative to ascertain whether these results can be applied to acute stroke care to guide subsequent acute therapeutic interventions.

## Data availability statement

The original contributions presented in the study are included in the article/supplementary material, further inquiries can be directed to the corresponding author.

## Ethics statement

The studies involving humans were approved by Ethics Committee of the Faculty 11, Medicine, Justus-Liebig University, Giessen, Germany. The studies were conducted in accordance with the local legislation and institutional requirements. The participants provided their written informed consent to participate in this study.

## Author contributions

TD: Methodology, Supervision, Writing – original draft, Writing – review & editing, Resources. LO: Methodology, Writing – original draft, Writing – review & editing, Data curation, Formal analysis, Validation, Visualization. TM: Data curation, Formal analysis, Writing – review & editing, Software. OA: Data curation, Writing – review & editing, Validation. US: Writing – review & editing, Methodology, Supervision. MJ: Methodology, Writing – review & editing, Project administration. HH: Project administration, Writing – review & editing, Resources, Supervision. SG: Project administration, Supervision, Writing – review & editing, Formal analysis, Methodology, Validation, Visualization, Writing – original draft.
